# Exploring drying pattern of a sessile droplet of genomic DNA in the presence of hematite nanoparticles

**DOI:** 10.1038/s41598-018-24821-1

**Published:** 2018-04-20

**Authors:** Rekha Bhar, Gurpreet Kaur, S. K. Mehta

**Affiliations:** Department of Chemistry, Panjab University, Chandigarh, India

## Abstract

For the first time, drying pattern of a sessile droplet of genomic DNA, in the presence of hematite nanoparticles was sighted by polarizing optical microscopy (POM) in this research article. POM results indicated that only at an appreciably high concentration of hematite nanoparticles dried pattern of deoxyribonucleic acid from calf thymus (CT-DNA) was altered. Iron hybridized cetylpyridinium chloride was utilized for the preparation of iron oxide nanoparticles through hydrothermal method. Fourier transforms infrared spectroscopy (FTIR) and powder x-ray diffraction (PXRD) studies confirmed the formation of highly crystalline hematite i.e. α-Fe_2_O_3_ nanoparticles. Morphology of the synthesized nanoparticle was visualized by transmission electron microscope (TEM) and field emission scanning electron microscope (FESEM), which revealed that nanoparticles were rhombohedral in shape with a size of 45 ± 10 nm. Based upon all the findings, hydrothermal growth mechanism was also proposed having bilayer protection of surfactant around the nanoparticles. UV-Vis spectroscopy and fluorescence spectroscopy were explored to study the affinity of thus prepared nanoparticles towards calf thymus deoxyribonucleic acid (CT-DNA). The low value of binding constant calculated from the spectroscopy data confirmed the weak interaction between nanoparticles and the CT-DNA.

## Introduction

The integration of biology and nanomaterials provide enormous opportunities to revolutionize many fields of science and technology. In particular, assimilation of nanoparticles (NPs) and deoxyribonucleic acid (DNA) holds notable position, owing to its importance in gene delivery, gene profiling, surface recognition, self-assembly/microarray, patterning etc.^1–3^. The genomic DNA contains thousands of genes, but not all the genes expressed together at one time. The two-dimensional microarray of DNA on a solid surface holds promising importance in the field of measuring the expression levels of a large number of genes together. Analysis of gene expression provides scientist an access to understand genomic hybridization in the closely related organism and to compare healthy and diseased cells profiles^4^. Drying DNA droplet attracts considerable attention as a simple methodology for generating DNA microarray. Study of dried droplets gains momentum after the exclusive investigation of the mechanism behind coffee ring stain formation by Deegan^5^. The pattering of drying droplets depends upon a number of key factors like concentration, the substrate surface, drying conditions etc. In the same context, Wong *et al*.^6^ studied the pattern of dried DNA droplet with an argument that when the concentration of DNA is sufficiently high, DNA gets drifted towards the periphery and forms DNA lyotropic liquid crystals. Rafailovich *et al*.^7^ also investigated the concentration depended behavior of DNA drying through confocal microscopy.They argued that, in case of high concentration, shell formation took place whereas, low concentration leads to the formation of an island. Progressively, drying patterns of DNA-NPs binary mixtures were also studied, however, the reports are scarce. Dynamic segregation of negatively charged DNA in the presence of negatively charged silica colloids was explored by Yoshikawa and co-workers^8^. They observed noticeable patterning of the DNA-colloid binary sessile drop as a function of DNA/silica colloid ratio. In another report, the effect of the size of silica nanoparticle on the DNA drying pattern was thoroughly examined by Zhu *et al*.^9^. They reported that 50 nm sized particles do not alter the drying pattern but when the size increased up to 2.89 µm, smoothed ring pattern becomes coarse. With advancement, Dugas and co-workers^10^ tried to manufacture DNA chips by exploring the droplet evaporation phenomena. However, due to the availability of limited research work, the area demands sincere attention to explore the DNA drying pattern. Effect of the presence of different types of NPs (metal NPs, metal oxide NPs, quantum dots etc.), shapes effect, concentration-effect drying process, initial DNA concentration, pH of suspension on the drying pattern of CT-DNA must be scrutinized.

Patterning of genomic DNA through drying or DNA microarray has importance in gene expression profiling, superhydrophobic surfaces, oriented surfaces etc. In the present report, an approach has been made to study the drying pattern of DNA in presence of iron oxide NPs, obtained from iron decorated cetylpyridinium chloride. Amongst the various available methods for the synthesis of the hematite nanoparticles (NPs), the hydrothermal method is exclusive, because it provides single step procedure for the synthesis of high product purity with narrow particle size distribution or homogeneity^11–13^. Amid different forms of iron oxide, hematite (α-Fe_2_O_3_) has distinguished features owing to its low cost, high resistance to corrosion, nontoxicity and environment friendliness^14,15^. Numerous reports are available on hydrothermal synthesis of hematite NPs but there is no report on the synthesis of hematite NPs from metal decorated surfactants. Metal decorated surfactant garnered sufficient attention as a two in one precursor for the synthesis of metal/metal oxide NPs. Mehta *et al*.^16,17^ elaborately described the role of transition metal functionalized surfactants and successfully employed the copper functionalized surfactant for the fabrication of air-stable copper NPs.

## Results and Discussion

### Physicochemical characterization

**Figure 1:**
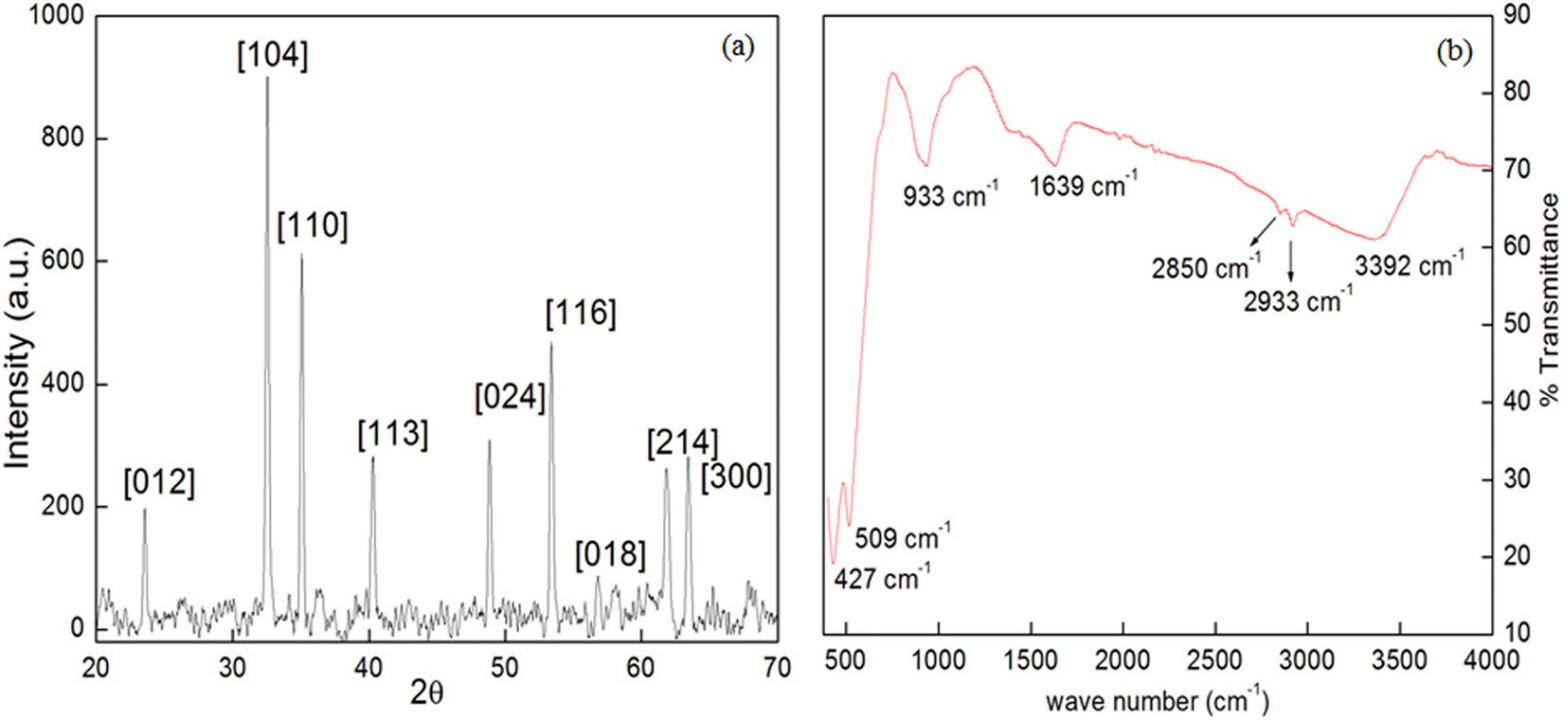
(**a**) PXRD pattern and (**b**) FTIR spectrum of synthesized material.

**Figure 2:**
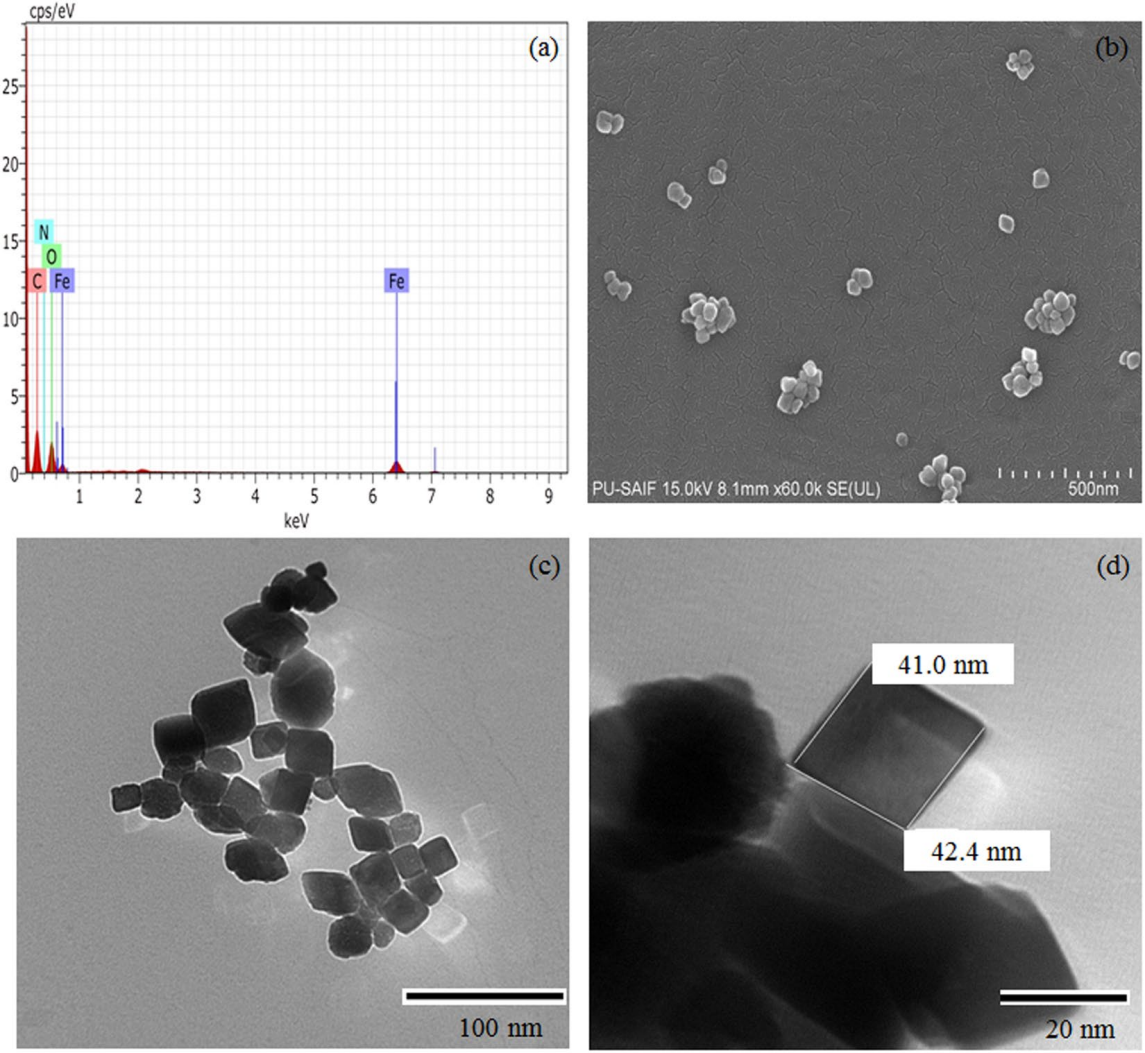
(**a**) EDS spectrum, (**b**) FESEM micrograph and (**c**,**d**) TEM images of the synthesized material.

Iron can form various types of oxides in II and III oxidation state. To confirm which form of iron oxide was formed, a number of characterization techniques were employed. However, the first evidence came from the color of the final product i.e. dark red, indicating that Fe_2_O_3_ (iron (III) oxide) was formed. Next task was to resolve that out of various Fe_2_O_3_ phases i.e. alpha, beta, and gamma which phase was possessed by synthesized NPs. Presence of Fe-O stretching frequency, at 427 and 509 cm^−1^ in the FTIR spectrum (Fig. 1b) confirmed the formation of iron oxide^18^. The vibrational frequencies at 933 cm^−1^, 1639 cm^−1^, 2933 cm^−1^, 3392 cm^−1^ ascribed to the -CH bending, -CH_2_ symmetric stretching, -OH stretching respectively, giving the evidence of the presence of surfactant coating on the NPs surface^19^. However, it is difficult to confirm the phase of Fe_2_O_3_ from FTIR. Then, size and phase composition of the synthesized material was determined by powder X-ray diffraction (PXRD). Observed PXRD pattern (Fig. 1a) revealed the formation of pure and homogeneous rhombohedral shaped hematite (α-Fe_2_O_3_) NPs, with no extra diffraction peak corresponding to other phase, was observed. Crystallite size was calculated by well-known Scherer formula, and it comes out to be 36.5 nm. Miller indices (hkl values) corresponding to 2θ and d spacing has also been tabulated in Table S1. FESEM and TEM micrograph (Fig. 2b,c and e) showed that particles were rhombohedral in shape with a size of 45 ± 10 nm. EDS coupled with FESEM confirmed the presence of iron, oxygen, carbon and nitrogen in the spectrum thus; supporting the argument of formation of surfactant coated Fe_2_O_3_ NPs (Fig. 2a).

Magnetic character of the Fe_2_O_3_ NPs was studied on vibrating sample magnetometer. M-H loop analysis revealed weak ferromagnetic hysteresis of NPs at room temperature with a saturation magnetization value of 0.00347 emu/g, which is one of the properties of α-Fe_2_O_3_ NPs. The low value of coercitivity (C_r_) and retentivity (M_r_) i.e. −1.72 × 10^−6^ and 8.76 × 10^−4^ also specified that synthesized iron oxide NPs are soft magnetic materials. Low saturation magnetization value as compared to the bulk material may be due to the presence of surfactant coating on Iron oxide NPs which act as a dead surface layer^15^.

### Plausible mechanism of hematite NPs synthesis

**Figure 3:**
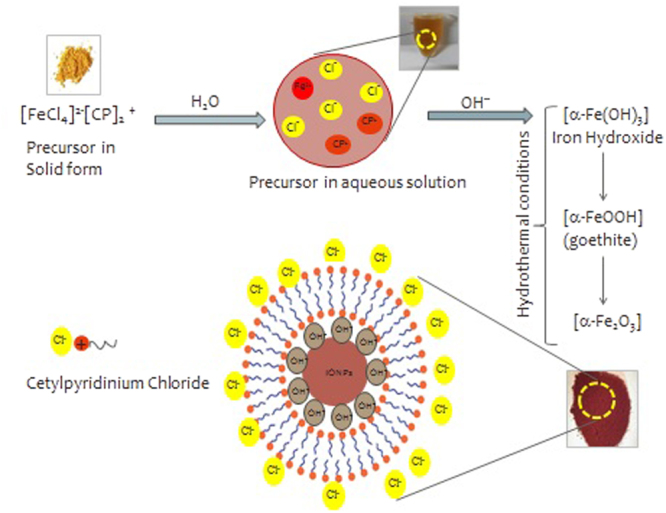
Mechanistic view of the synthesis of hematite NPs and capping by CPC.

After confirming the morphology, size, phase of the synthesized α-Fe_2_O_3_, an effort was made to understand the synthesis procedure mechanistically. In hydrothermal treatment, water acts as a catalyst or possesses different properties as its dielectric constant increases sharply above 100 °C and 1 atm. pressure. Fecpc II precursor dissolved in water generates Fe^+2^ ions which in the presence of hydroxyl ion forms iron (II) hydroxide. Subsequently, under the hydrothermal condition and basic media iron II hydroxide get oxidized to iron (III) oxy-hydroxide i.e. FeOOH (goethite) by the dissolved oxygen in an aqueous medium. Finally, dissolution of goethite leads to the precipitation of Fe_2_O_3_ nanomaterial^20,21^. To understand the role of CPC in synthesis, thermogravimetric studies (TGA) were performed. TGA of pure CPC and CPC coated NPs were recorded. Three weight loss steps were visible in TGA curve of CPC coated NPs while only two in pure CPC (Supplementary Fig. S1(a,b)). The first step corresponds to the loss of loosely held molecules like water, carbon dioxide etc. in both the samples. The second step in TGA curve of pure CPC is quite sharp, which indicates that CPC gets decomposed in a single step within in the temperature range of 220–270 °C. However, CPC in capped NPs behave somewhat differently and that could be attributed to the fact that it may be arranged as a bilayer around the hematite NPs (Fig. 3). Bilayer formation would be easily justified, as in aqueous basic media hematite NPs may acquire OH^−^ ions (adsorbed OH^−^ ions on NPs as revealed by FTIR spectrum) on its surface which then attracts the positively charged head group of CPC keeping the hydrophobic tail in the aqueous medium.

Then, another layer of CPC gets arranged with its head group facing aqueous medium and tail towards tail part of the first layer to make the system stable through hydrophobic interaction. The second and third weight loss step in CPC coated NPs is due to the loss of outer layer and an inner layer of CPC respectively^22^. To further validate the phenomena, EDX mapping was carried out. The images clearly demonstrate the quantitative distribution of the elements present in the sample (Fig. S4). A close look of mapping image revealed that the relative position or distribution of elemental nitrogen in pairs. This can be correlated with that of the proposed mechanism of bilayer capping of cetylpyridinium chloride on hematite nanoparticles. Also, the density of elements present in the sample as shown in the Fig. S4 is in good agreement with the % quantity of elements from EDX (Table S2).

### CT-DNA interaction studies

#### Absorption studies

**Figure 4:**
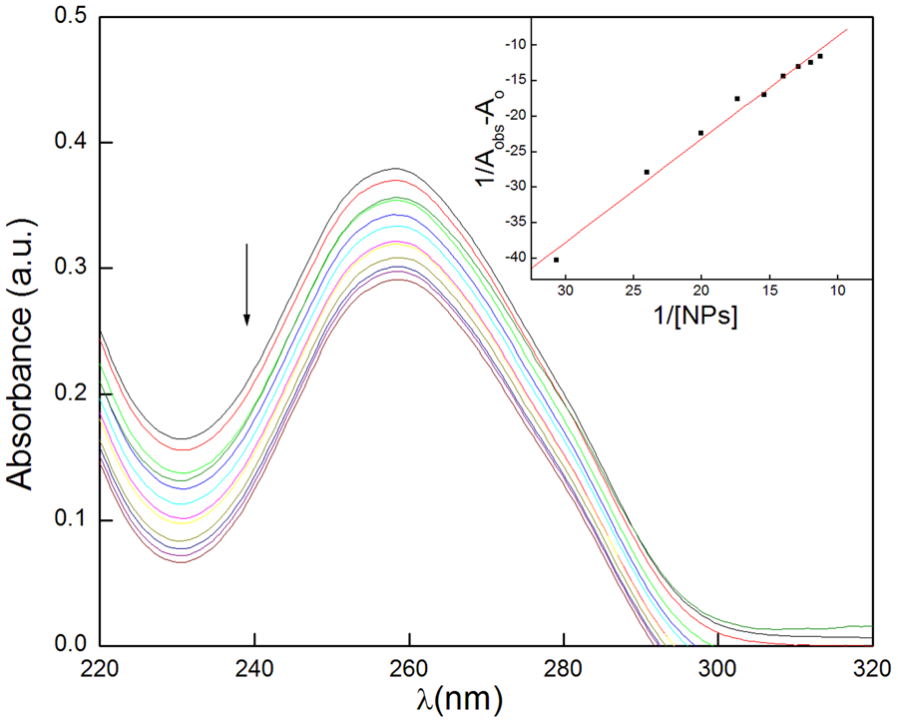
UV-vis spectra of CT-DNA with increasing concentration of hematite NPs (inset of the figure is B-H plots of the corresponding data).

Strong absorption by nitrogenous bases present in CT-DNA provides an easy access to detect the binding of small molecules with CT-DNA through UV-vis spectroscopy. Absorption spectra of CT-DNA (50 µM) were recorded at varying the concentration of hematite NPs (Fig. 4). The absorption spectrum of only hematite NPs was also recorded (Supplementary Fig. S2). It is clear from the Fig. 4 that, as the concentration of hematite NPs increases absorption intensity decreases with no shift in the λ_max_. Observed hypochromism is indicative of the electrostatic or intercalative mode of interaction with CT-DNA^23^. Intercalation mode is less probable as hematite NPs do not have a planar conjugated aromatic group in it, which can interact through π-π stacking mode^24^. At a first glance, the chance of electrostatic mode of interaction also seems less probable as hematite NPs do not carry any surface charge (zeta potential value approx. zero). However, weak interaction forces can be developed between polar/charged CT-DNA and uncharged hematite NPs through dipole-induced dipole mechanism. To further validate this point, the absorption data has been analyzed by Benesi-Hildebrand equation^25^:

1 $$\frac{1}{{A}_{obs}-{A}_{o}}=\frac{1}{{A}_{c}-{A}_{o}}+\frac{1}{{K}_{app}({A}_{c}-{A}_{o})[NPs]}$$

Here, A_obs_, A_o_, A_c_ are the observed absorbance after the addition of hematite NPs, of pure CT-DNA, hematite NPs and CT-DNA mixture at equilibrium, respectively. K_app_ is the apparent binding constant and [NPs] is the concentration of hematite NPs. The double reciprocal graph viz. B-H plot between 1/A_obs_ − A_o_ and 1/[NPs] (inset of the Fig. 4) depicts the linearity with regression value of 0.98. K_app_ can be calculated from the slope of B-H plot, which is equal to the 1/K_app_(A_c_ − A_o_).The value of K_app_ comes out to be 7.91 L/g. This magnitude is well coordinated with the weak interactive forces.

### Competitive EtBr binding studies

**Figure 5:**
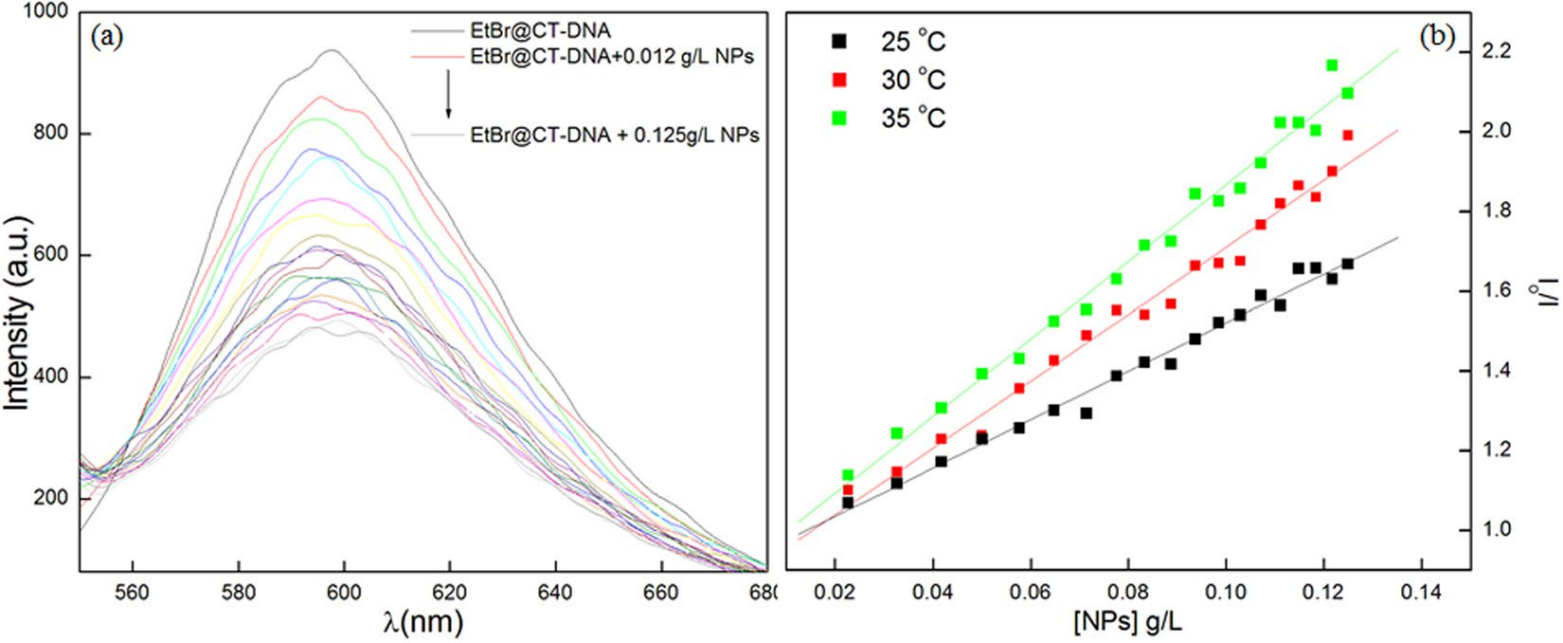
(**a**) Fluorescence spectra of EtBr @CT-DNA at different concentration of hematite NPs at 25 °C (Arrow indicate change in intensity with increase in hematite NPs concentration). (**b**) Stern Volmer plots at the different temperature.

Though DNA does not give fluorescence, yet interaction of small molecules with DNA can also be studied through emission spectra. Emission studies with DNA can be possible using a fluorescent probe i.e. EtBr. Upon intercalation with DNA, the fluorescence intensity of EtBr get enhanced significantly^26^. Figure 5 shows fluorescence of EtBr intercalated CT-DNA at different concentration of hematite NPs at 25 °C. With the increase in the concentration of hematite NPs, fluorescence intensity decreases gradually and after that, no significant change was observed. The decrease in the intensity may be attributed to either complete dislocation or slight displacement of EtBr from CT-DNA. Complete dislocation of EtBr from CT-DNA can be ruled out as explained earlier. Small moieties get fit into grooves of DNA and this slightly displaces the EtBr from CT-DNA resulting in a decrease in the fluorescence intensity^27^. Binding strength in such cases would be very less. To justify the hypothesis, fluorescence data was evaluated by Stern-Volmer equation^28^:

2 $$\frac{{I}_{0}}{I}={K}_{sv}[NPs]+1$$

Here, I_o_, I, K_sv_, [NPs] are the intensity of EtBr@CT-DNA only, in the presence of hematite NPs, Stern Volmer constant, and concentration of hematite NPs, respectively.To know the mechanism of interaction, fluorescence was recorded at three different temperatures. The K_sv_ values at all the temperature under investigation, have been tabulated in Table 1. Two main conclusions can be drawn, firstly low magnitude of Stern Volmer constant indicating weak forces of interaction between hematite NPs and CT-DNA. Secondly, fluorescence quenching may be due to the collision, as with the increase in temperature, Stern Volmer constant value also increased^29,30^. With the increase in temperature, the mobility of the interacting moieties increases hence, more chances to get into the proximity of each other resulting in higher Stern Volmer quenching constant value.

**Table 1:** K_sv_ values at different temperatures with corresponding regression (R) value.

T (K)	K_sv_ (L/g)	R^2^
298.15	7.71	0.99
303.15	8.45	0.99
308.15	8.78	0.99

### Thermodynamic parameters and interaction forces

Weak interactive forces may include hydrophobic interaction, electrostatic or van der Waal forces. To confirm, fluorescence data can be further taken into consideration to know about the thermodynamics of the system under study. Since the range of temperature study is small (enthalpy change considered constant), data can be evaluated by Vant Hoff equation^29^:

3 $$lnK=-\,\frac{{\rm{\Delta }}H}{RT}+\frac{{\rm{\Delta }}S}{R}$$

Here, K is taken analogs to the binding constant at different temperatures (T) and R is the universal gas constant. The values of ΔH and ΔS were calculated from the slope and intercept of the graph between lnK and 1/T respectively and have been tabulated in Table 2. The spontaneity of the interactive forces between hematite NPs and CT-DNA has also been anticipated from the free energy change (ΔG) values (Table 2) using following equation:

4 $${\rm{\Delta }}G={\rm{\Delta }}H-T{\rm{\Delta }}S$$

**Table 2:** Various thermodynamic parameters of hematite NPs and CT-DNA interacting system.

T(K)	ΔG (kJ/mg)	ΔH(kJ/mg)	ΔS (J/mgK)
298.15	−5.06	−8.52	−11.61
303.15	−5.37		−12.46
308.15	−5.47		−12.57

The negative values of free energy change affirmed that interaction between hematite NPs and CT-DNA is favorable. Further, negative values of ΔH and ΔS indicate that the weak van der Waals forces play the major role in the present system^29^. Absorption and emission studies collectively concluded that affinity of hematite NPs towards CT-DNA is not strong, however, such weak interaction also found application in the various scientific arena and holds a significant interest in the biomedical field. The weak interactive forces between NPs and CT-DNA are similar to repressor protein-DNA interaction *in vivo*, which is the prime requisite for the gene therapy where binding of DNA macromolecule to the carrier vehicle is an important aspect^31^. Such binding may be either on the surface or inside the domain of carrier. But these bindings must be weak in nature in order to intracellular release of the genetic material^32,33^.

### Evaporative self-assembly of CT-DNA and hematite NPs binary mixture

**Figure 6:**
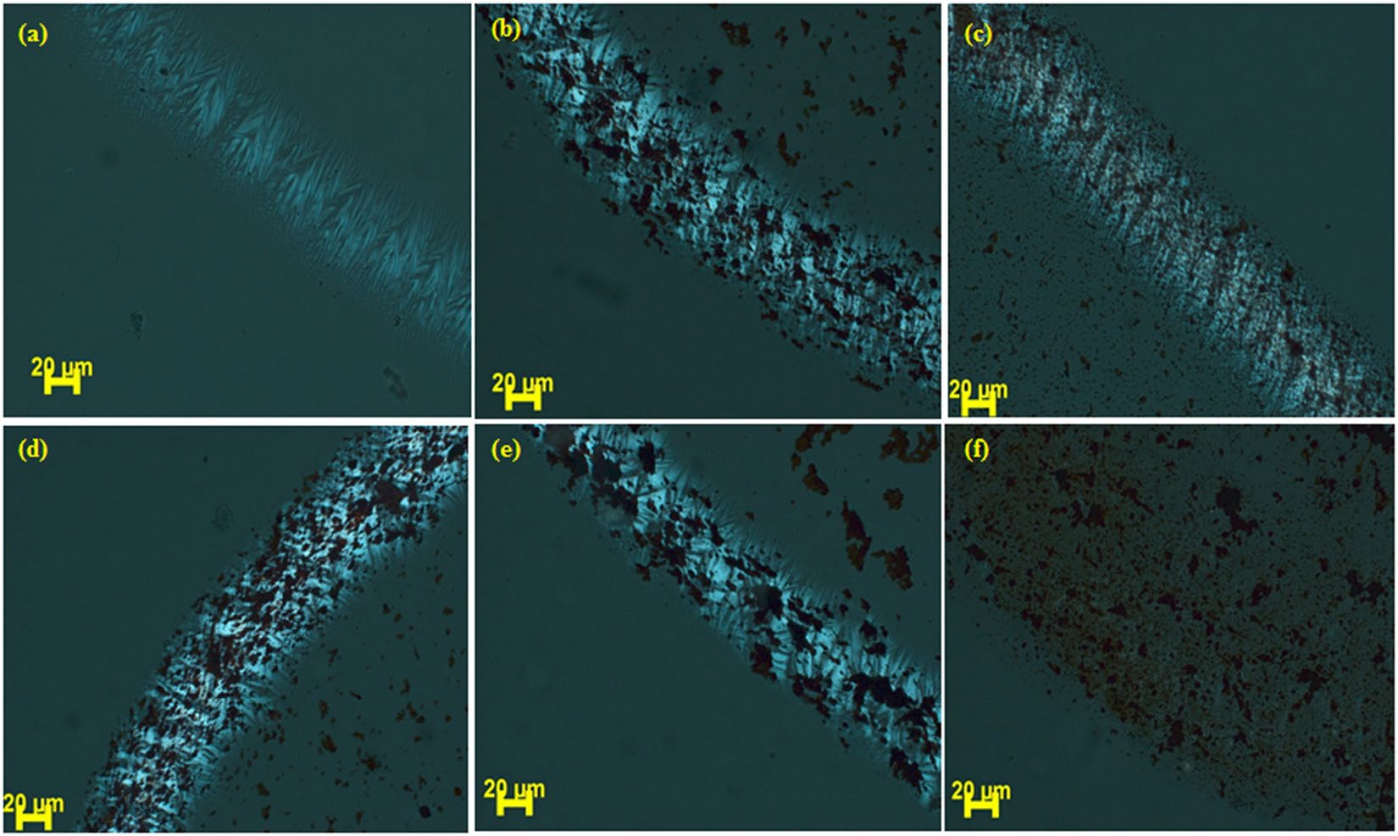
POM images. (**a**) Top view of CT-DNA only, (**b**–**f**) CT-DNA with hematite NPs 2.5 mg/ml, 5 mg/ml, 10 mg/ml, 20 mg/ml, 50 mg/ml respectively.

**Figure 7:**
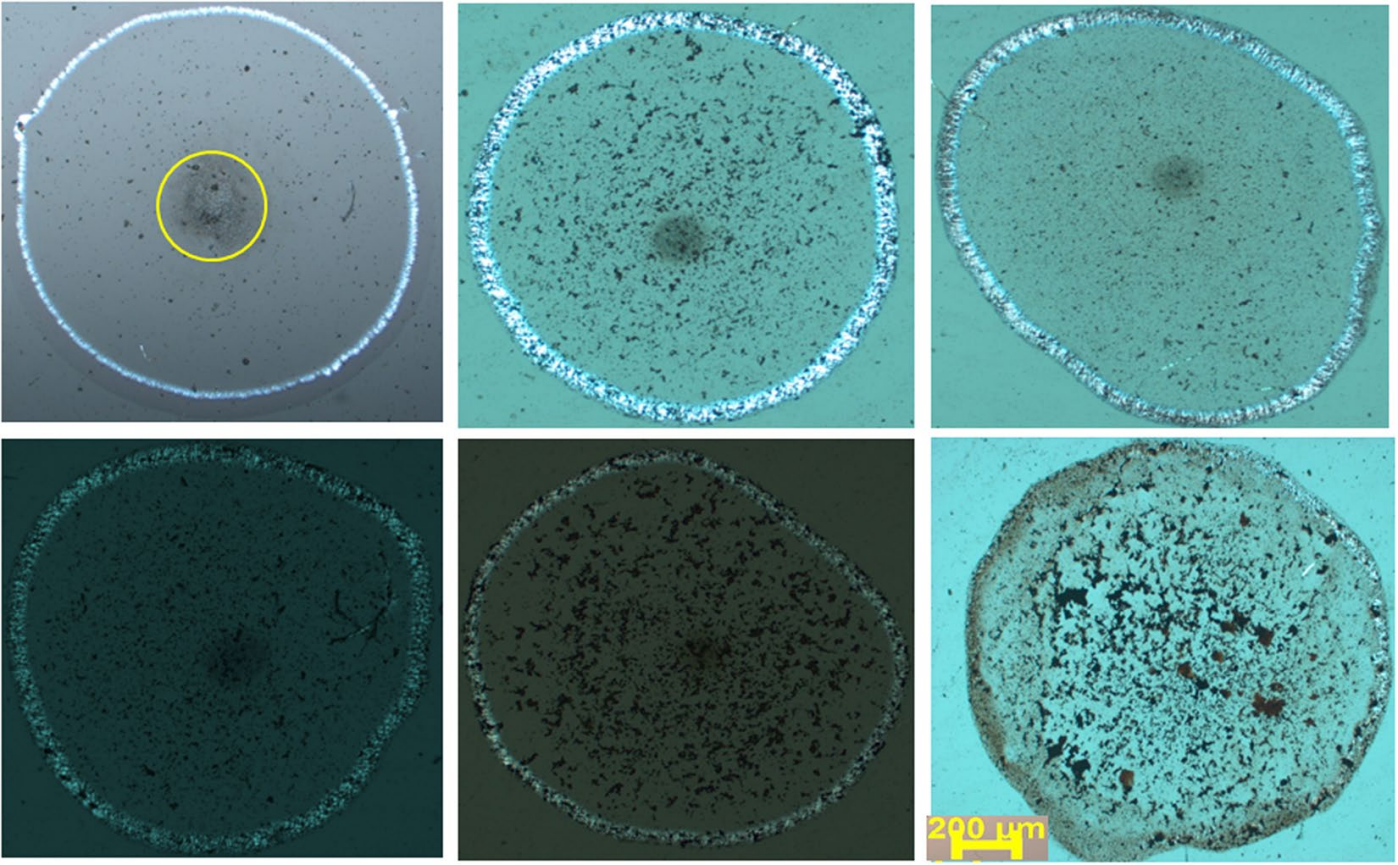
POM images. (**a**) Top view of CT-DNA only, (**b**–**f**) CT-DNA with hematite NPs 2.5 mg/ml, 5 mg/ml, 10 mg/ml, 20 mg/ml and 50 mg/ml respectively. All images are at same scale as mentioned in (**f**).

The affinity of synthesized hematite NPs with CT-DNA was also investigated by analyzing the drying pattern of CT-DNA in the presence of hematite NPs. To the best of our knowledge, there is no research article which describes the effect of hematite NPs on drying pattern of DNA. In the present article, drying droplet of CT-DNA and its mixture with hematite NPs were explored by POM. A concentrated solution of CT-DNA in water was prepared and a sessile drop was dried on cleaned glass cover slip. Figure 6 (peripheral view of the dried pattern) and 7 (complete view of the dried pattern) showed the pattern of the dried droplet which indicated that CT-DNA got drifted towards the periphery and birefringent lyotropic liquid crystals were formed. No patternization was observed in the case of hematite NPs alone (Fig. S3). Thus formed zigzag periodic pattern is in contrary to the typical coffee ring stain drying mechanism, where transport of the material to the periphery did not happen. Wong *et al*.^13^ thoroughly explained this phenomenon on the basis of models for liquid crystal elasticity. After visualizing the Figs 6 and 7, it can be said that low concentration of hematite NPs does not affect the birefringent lyotropic liquid crystalline phase of CT-DNA. When the concentration of hematite NPs was appreciably high, we observed disturbance in drying pattern (Fig. 6(f)) of CT-DNA. This might be due to the fact that higher concentration of hematite NPs did not allow peripheral movement of CT-DNA. Another important aspect shown by POM images was that hematite NPs can be seen under the microscope as red-colored aggregated lumps. A slight aggregated form of hematite NPs can be seen in TEM and FESEM images, however, evaporation of the liquid in the sessile droplet, facilitate the aggregation further and thus making it possible to visualize nano-ranged moiety under a microscope. Figure 7 also showed that there was also lied a different area in the dried pattern (marked in a circle). Detailed analysis of POM images revealed that not only the peripheral region but also the center of the dried pattern has distinguished pattern altogether (Supplementary Fig. S5). As explained earlier, CT-DNA got drifted towards periphery then, what else can form the pattern? After careful analysis of these patterns, we anticipated that these are tiny water droplets which remained wet even after drying process^34^. To confirm, the drying process for a longer period of time was tried and it was observed that up to 6 six hours of drying, the pattern remained the same. The sample was then kept overnight for drying under a bulb. Unfortunately, instead of getting the completely dried pattern, the lyotropic crystals pattern that observed earlier gets completely disturbed due to the arbitrary orientation of the molecules at higher temperature^35^. We believed that the complete dryness cannot be achieved along with the said pattern. But in Fig. 7(f) this pattern is absent confirming that droplets with 50 mg/ml hematite NPs dried completely within the same duration i.e. within 4 hr. This might be due to the adsorption of excess water onto the surface of NPs.

Absorption and emission studies stated that hematite NPs have a very low affinity towards the CT-DNA. This is further assured by POM studies, as only very high concentration of hematite NPs could have affected the drying pattern of CT-DNA.

To conclude, in the present report, a simplistic method for the synthesis of hematite NPs was successfully developed. Hydrothermal treatment of the iron decorated metallosurfactant results in the assortment of highly crystalline, rhombohedral shaped hematiteNPs having amphiphilic protection on their surface for better size control. Interaction of the synthesized nanoparticle with CT-DNA was assessed through UV-vis spectroscopy, fluorescence spectroscopy, and POM. The value of the binding constant in the range of 7.91–8.78 L/g from different studies indicated that the weak forces of interaction plays the major role between CT-DNA and hematite NPs. Thermodynamic data also gave weightage to the same findings. A thorough analysis of POM images indicated that drying pattern of CT-DNA i.e. formation of lyotropic liquid crystals upon drying of a sessile drop of CT-DNA in a controlled environment was disturbed, only at a very high concentration of hematite NPs. Such findings open up window to investigate further, the drying pattern of CT-DNA in presence of different type of NPs, solvents, different drying process, at different ionic strength (pH studies) for applications like control wetting, oriented surfaces, printing, genotyping etc.

## Experimental

### Chemicals and Materials

Ferrous Chloride (99.0%), Cetylpyridinium Chloride monohydrate (CPC.H_2_O, 99.0–102.0%), Ethidium Bromide (EtBr, ~95%), Sodium hydroxide (99.9%) and Deoxyribonucleic acid from calf thymus (CT-DNA) type XV, activated, lyophilized powder were obtained from Sigma Aldrich. Information regarding melting temperature, absorbance i.e. λ_max_, % base pairs, preparation instructions and storage precaution for CT-DNA have been provided in the supplementary information. Sodium dihydrogen phosphate (≥99.0%), Disodium hydrogen phosphate (≥99.0%) were from Merck. Acetone for synthesis was purchased from Fischer Scientific. All chemical used in the present study were of high purity and used as such without further purification.

### Synthesis of NPs

Hematite (α-Fe_2_O_3_) NPs were synthesized hydrothermally from iron cetylpyridinium chloride II (FecpcII) precursor. Fecpc II was synthesized by the previously known method^36^. The pH of an aqueous solution of 20 mM Fecpc II was adjusted to 9.0 by NaOH. The solution was then transferred to a Teflon lined autoclave and kept at 130 °C for 5 hours. The final product (red-brown in color) was collected by centrifugation and then washed thoroughly with water and acetone to remove the unreacted reactants. Lastly, the desired nano-powder was obtained by drying the washed product under vacuum.

### Instrumentation

FTIR spectrum was recorded on Nicolet iS50 FT-IR instrument from Thermo Scientific. Morphology of the synthesized NPs was visualized through transmission electron microscopy (TEM) and Field emission scanning microscopy (FE-SEM) on Hitachi H-7500 and Hitachi-SU8010 microscope respectively. Elemental analysis of the product was resolute from EDS coupled with FESEM instrument. Images of the dried pattern were captured using Carl ZEISS Axio Imager.2Am microscope. Captured images were analyzed by axio-vision software.

### Absorption and Emission Studies

Absorption spectra of CT-DNA in the presence of hematite NPs were recorded on HALO DB-20 UV-Vis double beam spectrophotometer from Dynamica. The concentration of CT-DNA was kept at 50 µm and concentration of hematite NPs varied until saturation point was reached. Spectra were recorded at room temperature. To avoid the interference of NPs, the baseline correction was done by adding an equal amount of hematite NPs to the reference as well as the sample cell.

Fluorescence studies were carried out on Hitachi F-7000 FL spectrophotometer. An excitation wavelength of 490 nm was given to samples and an emission range of study was chosen to be 550–650 nm. Excitation and emission slit width was set at 10 nm, with a PMT voltage of 700 V. CT-DNA concentration was kept constant at 10 µM and concentration of synthesized hematite NPs varied from zero to 125 µg/ml. The concentration of hematite NPs was gradually increased by adding a 100 µl aliquot of a stock solution of concentration 0.25 mg/ml of hematite NPs. All the studies were carried out in phosphate buffer of pH 7.4. The same set of experiment was conducted at three different temperature.

The absorption and emission of blank buffer solution were also recorded and subsequently subtracted from the absorption and emission spectra of the sample.

### Preparation of DNA solution/ DNA and NPs binary mixture

A viscous solution of CT-DNA was prepared by dissolving 5 mg CT-DNA in 1 ml of double distilled water. The solution was then kept at 4 °C for 24 hr. or until a clear solution was obtained. To understand the behavior of DNA in the presence of hematite NPs, a series of samples were prepared by keeping the concentration of DNA same and varying the concentration α-Fe_2_O_3_ NPs. Then, 2.5 µl aliquot of the DNA solution was mixed with synthesized NPs at various concentrations. Final, volume was adjusted to 10 µl by adding double distilled water and incubated for half an hour at room temperature. Thus obtained solution was used as such for studying drying pattern of DNA in the presence of hematite NPs.

### Preparation of slides for microscopy

An aliquot of 2.5 µl of the thus prepared mixture was put on a thoroughly cleaned glass coverslip and dried slowly at ambient temperature under a blub for 4 h. Two different types of samples, one with CT-DNA only and other with hematite NPs only as control samples were also dried under the same conditions.

## Electronic supplementary material

Supplementary Information
