# Supplementary material for: Exploring drying pattern of a sessile droplet of genomic DNA in the presence of hematite nanoparticles
Source: Sci Rep. 2018 Apr 20;8:6352. doi: 10.1038/s41598-018-24821-1 (PMC5910388; doi:10.1038/s41598-018-24821-1)
Supplement: Supplementary file 1 — Supplementary Information [file 41598_2018_24821_MOESM1_ESM.docx]

**Supplementary Information**

Exploring drying pattern of a sessile droplet of genomic DNA in the presence of hematite nanoparticles

Rekha Bhar, Gurpreet Kaur, S.K. Mehta*


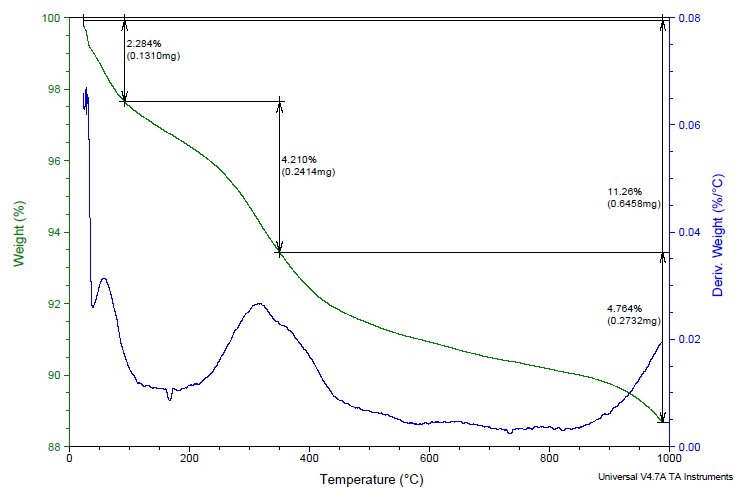

Figure S1 TGA curve of (a) CPC coated hematite nanoparticles and (b) pure CPC.

(a)

(b)

Figure S2 UV-vis spectrum of synthesized iron oxide nanoparticles.


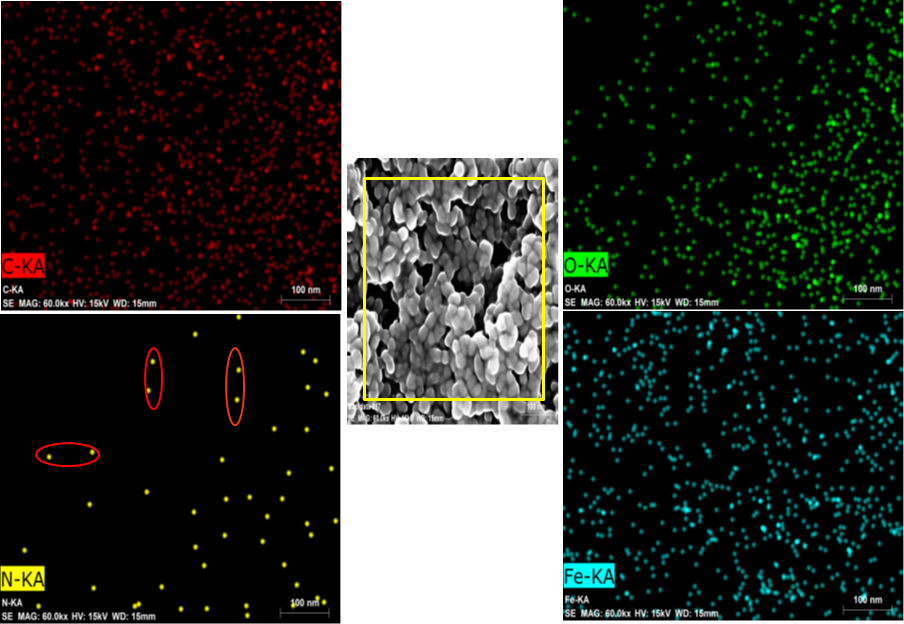


Figure S3 POM images of hematite NPs at different magnification.


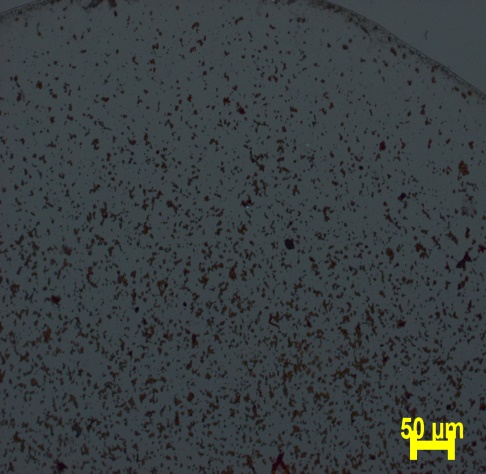

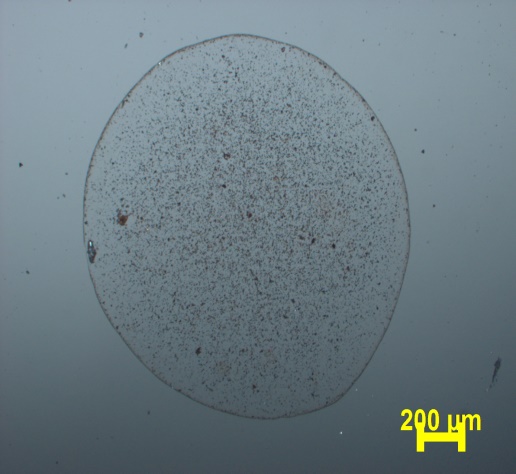

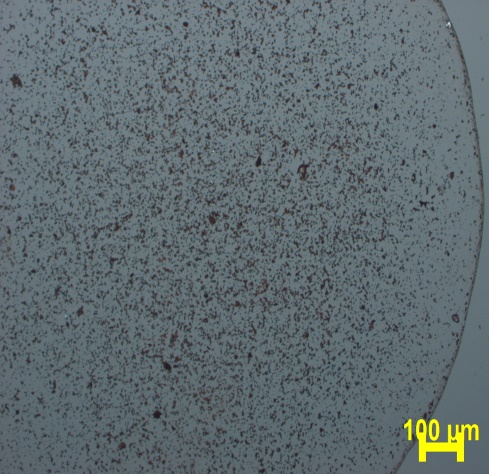


Figure S4 EDX mapping images of the synthesized hematite nanoparticles.

Table S2 Elemental composition of hematite nanoparticles from EDX analysis.


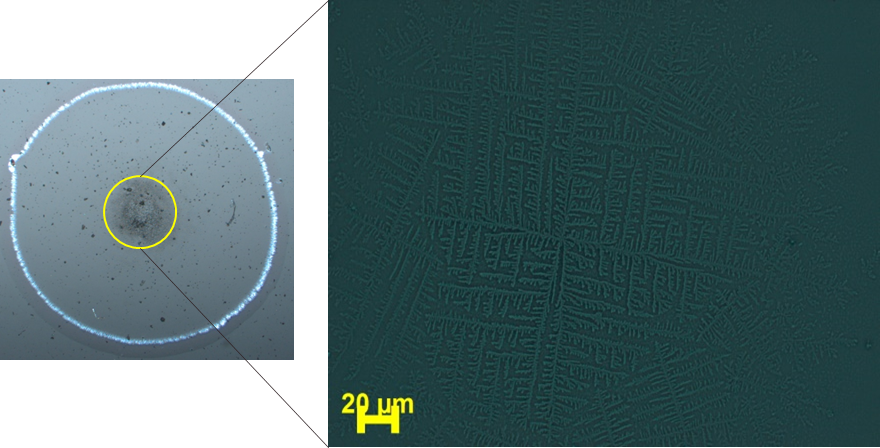


Figure S5 Enlarged view of the central part of dried droplet of DNA.

| Element | Wt. % |
| --- | --- |
| Carbon | 38.49 |
| Oxygen | 30.74 |
| Iron | 27.18 |
| Nitrogen | 3.59 |

Table S1. The hkl plane values and d-spacing to the corresponding 2 θ values from X-ray diffraction of hematite NPs

| **2θ** | **d-spacing** | **h k l plane** |
| --- | --- | --- |
| 23.77 | 3.77 | 012 |
| 32.59 | 2.74 | 104 |
| 35.11 | 2.55 | 110 |
| 40.57 | 2.22 | 113 |
| 48.79 | 1.85 | 024 |
| 53.35 | 1.71 | 116 |
| 57.57 | 1.59 | 018 |
| 62.19 | 1.49 | 214 |
| 63.66 | 1.46 | 300 |

**CT-DNA Specification**

Melting Temperature: 87 °C

λ_max_: 259 nm (100 mM phosphate buffer, pH 7.0)

DNA from calf thymus is 41.9 mole % G-C and 58.1 mole % A-T. An absorbance of 1.0 at 260 nm corresponds to approximately 50 mg of double-stranded DNA. To prevent shearing of the large genomic DNA, this product should be dissolved in water (1 mg/ml) with no sonication or stirring. Gentle inversion overnight at 0 - 4 °C is recommended to completely solubilize the DNA.

Preparation Instructions

The lyophilized DNA should be reconstituted overnight at 2–8 °C to insure that all the material goes into solution. When the product is fully dissolved, the DNA concentration can be determined by measuring the absorbance at 260 nanometers (A260) and using the following formula: mg/ml of DNA = A260 x 50 mg/ml x DF A260 = absorbance of the DNA solution at 260 nm 50 mg/ml = the concentration of 1 A260 unit of dsDNA DF = the dilution factor (typically a 100-fold dilution with reconstitution buffer for a 1 mg/ml solution)
